# Performance comparison and clinical validation of two rapid direct identification and antimicrobial susceptibility testing approaches for pathogens isolated from positive blood cultures in children

**DOI:** 10.3389/fcimb.2026.1861487

**Published:** 2026-09-17

**Authors:** Fen Pan, Fangyuan Yu, Tiandong Zhang, Qin Cai, Jie Jiang, Hong Zhang, Pengcheng Chen, Wenhao Weng

**Affiliations:** 1 Department of Clinical Laboratory, Shanghai Children’s Hospital, School of Medicine, Shanghai Jiao Tong University, Shanghai, China; 2 Institute of Pediatric Infection, Immunity, and Critical Care Medicine, Shanghai Jiao Tong University School of Medicine, Shanghai, China; 3 Department of Clinical Laboratory, Shuguang Hospital Affiliated to Shanghai University of Traditional Chinese Medicine, Shanghai, China

**Keywords:** bloodstream infections, clinical performance, positive blood culture, rapid antimicrobial susceptibility, rapid identification

## Abstract

**Objective:**

Rapid species identification and antimicrobial susceptibility testing (AST) for bloodstream infections (BSIs) are critical for timely and targeted therapy. This study evaluated the clinical performance of two rapid methods (HBL rapid method and rapid method with short-term culture) against the routine standard blood culture (BC) method.

**Methods:**

A total of 162 monobacterial BC samples were enrolled. These samples were processed either using the HBL system (subcultured within 2 hours) or incubated on agar plates for short-term culture (6-8 hours). Subsequent species identification and AST were also performed. Essential agreement (EA), categorical agreement (CA), and error rates were calculated as compared to the conventional method.

**Results:**

Among 162 isolates, Gram-negative bacteria comprised 66.7% (108/162) of the isolates, predominantly *Enterobacteriales*. Both the rapid methods demonstrated 100% species identification concordance with the standard BC method. In terms of AST, using the standard AST result as reference, the HBL rapid method yielded an EA of 95.6% and a CA of 98.3%, with minor error (mE), major error (ME), very major error (VME), and bias rates of 1.1%, 0.4%, 0.1%, and -2.3%, respectively. Similarly, the rapid method with short-term culture showed comparable efficacy (EA, 96.5%; CA, 98.8%; mE, 1.0%; ME, 0.2%; VME, 0.1%; bias, -0.3%). Crucially, the mean turnaround times (TATs) for the HBL rapid method (44.7 hours) and the rapid method with short-term culture (45.9 hours) were significantly shorter than the standard method (68.4 hours), representing an approximate 24-hour reduction in BC workflow.

**Conclusions:**

The two rapid methods exhibit exceptional accuracy and reliability for species identification and AST directly from positive blood cultures, which can significantly shorten the TAT for BSI diagnosis, offering clinicians to optimize timely and appropriate clinical treatment.

## Introduction

Bloodstream infections (BSIs) represent one of the most prevalent and life-threatening infectious syndromes globally, with persistently high morbidity and mortality despite remarkable advances in antimicrobial therapy and critical care. The global disease burden of BSIs is staggering, and contemporary epidemiological estimates indicate approximately 30-50 million cases annually, with associated crude mortality rates reaching 20-40% (Holmes et al., 2025; Rudd et al., 2020). In recent years, the global dissemination of multidrug-resistant organisms including carbapenem-resistant Gram-negative bacilli and methicillin-resistant *Staphylococcus aureus* has further exacerbated this crisis, driving up BSI-related mortality and severely constraining clinical antibiotic treatment options (Falcone et al., 2023; Kaku et al., 2025). Timely and accurate etiological diagnosis, along with early administration of targeted antimicrobial therapy, is a cornerstone of optimal BSI management; any delay in this step is consistently associated with poor clinical outcomes, including increased mortality, prolonged hospital stays, and elevated health costs.

Blood culture (BC) remains the gold standard for the microbiological diagnosis of BSIs, underpinning pathogen identification and antimicrobial susceptibility testing (AST) for target therapy. However, conventional BC workflows face profound limitations in the current era of rising antimicrobial resistance (AMR) and growing demands for rapid, patient-centered care. Notably, a critical drawback of automatic BC systems is the unacceptably prolonged time to results, with standard incubation requiring 24-72 hours for pathogen detection and an additional 16-24 hours of subculture needed to complete AST. To address this unmet clinical need, clinical microbiology laboratories worldwide have dedicated considerable efforts to developing and validating accelerated BC processing strategies over the past two decades, with the dual goals of shortening the turnaround time (TAT) and improving patient outcomes.

Notably, in terms of rapid pathogen identification, matrix-assisted laser desorption time-of-flight mass spectrometry (MALDI-TOF MS) has emerged as a transformative tool, enabling rapid, accurate, and cost-effective species-level identification results using centrifuged bacterial pellets from positive BC broths or short-term culture on solid media (Fitts et al., 2024; Karadag and Ergon, 2024). Similarly, advances in rapid AST(RAST) have sought to overcome the bottleneck of traditional AST, which relies on 16-24 hours of subculture before assessing bacterial growth in the presence of antimicrobial agents. Recognizing the clinical urgency of RAST, both the European Committee on Antimicrobial Susceptibility Testing (EUCAST) and the Clinical and Laboratory Standards Institute (CLSI) have issued formal recommendations for validated RAST methods, designed to shorten the detection time of AST for positive BC samples (Jonasson et al., 2023; Salian et al., 2025). Additionally, a growing array of commercial and laboratory-developed RAST platforms have been engineered for direct testing from positive BC samples, obviating the need for overnight subculture and further streamlining diagnostic workflows (Anton-Vazquez et al., 2019; Kim et al., 2024; Ostermann et al., 2024; Tibbetts et al., 2022).

The HBL system (Alifax, Polverara, Italy) is an automated liquid culture platform that leverages a proprietary light-scattering technology to detect bacterial growth turbidity with high sensitivity and speed. Building on this technology, we developed a modified BC workflow by directly using the HBL system for rapid cultivation in 2 hours to shorten the time of bacterial culture, followed by concurrent rapid pathogen identification and AST testing. As another rapid strategy, we also established a rapid method with short-term culture, whereby positive BC samples are incubated on blood agar plates for 6-8 hours prior to rapid identification and AST testing. To date, the diagnostic performance of these two laboratory-developed rapid workflows has not been systematically evaluated and compared with the conventional BC method in the pediatric population. In this study, we comprehensively assess the analytical and clinical performance of two rapid methods (the HBL rapid method and the rapid method with short-term culture) against the conventional BC workflow, which aims to optimize the diagnostic workflow for positive BC samples and address the critical limitation of prolonged result reporting in conventional BC protocols.

## Materials and methods

### Study design

The BC samples were collected from pediatric patients with suspected BSIs between June 2022 and August 2024 in the clinical microbiology laboratory of Shanghai Children’s Hospital. All samples were inoculated into BACTEC Peds Plus bottles and incubated on the Bactec FX400 automatic blood culture system (Becton Dickinson, Franklin Lakes, NJ, USA) until a positive growth signal was detected or for a maximum of 7 days of incubation. Subsequently, all positive BC samples underwent Gram staining to exclude polymicrobial infections, and only samples confirmed to have monobacterial growth were eligible for subsequent pathogen identification and AST. Some samples initially appearing monobacterial on Gram stain subsequently yielded polymicrobial growth on culture, which were excluded from the study. Finally, a total of 162 consecutive monobacterial positive BC samples were selected for this study and were processed in parallel using the two established rapid testing methods and the conventional standard BC workflow. A time-dependent testing algorithm was applied for sample processing as follows: for BC bottles flagged positive between 17:01 the preceding day and 11:00 on the testing day, both the HBL rapid culture method and the rapid method with short-term culture were performed simultaneously. For BC bottles flagged positive between 11:01 and 17:00 on the testing day, only the HBL rapid culture method was implemented because of laboratory workflow feasibility and time constraints for short-term solid-media incubation.

### Standard BC method

Processing of positive BC samples by the conventional standard method was performed as illustrated in **Figure 1**. Briefly, 500 μL aliquots from positive BC bottles were subcultured onto Columbia blood agar plates with 5% sheep blood and MacConkey agar plates (Yihua Co., Ltd., Shanghai, China) and incubated at 35 ± 1°C with 5% CO_2_ for 18-24 hours. Following incubation, identification of single colonies was performed by using MALDI-TOF MS (Bruker Daltonics, Bremen, Germany) in accordance with the manufacturer’s instructions. AST was subsequently carried out based on the identified bacterial species. For *Enterobacteriales*, *Pseudomonas aeruginosa*, and *Staphylococcus* spp., AST was performed using the Vitek2 automated susceptibility testing system (bioMérieux, Marcy l’ Étoile, France) with GN-334, GN-335, and GP-639 cards, respectively. In addition, AST of *Salmonella* spp., *Enterococcus* spp., and *Streptococcus* spp. was performed by the Kirby-Bauer disk diffusion method on Mueller-Hinton (MH) agar or MH agar supplemented with 5% defibrinated horse blood depending on bacterial species. However, the AST of penicillin, ceftriaxone, and amoxicillin/clavulanate for *Streptococcus pneumoniae* and penicillin for other viridans streptococci were measured by E-test methods. Standard strains including *Escherichia coli* ATCC 25922, *P. aeruginosa* ATCC 27853, *S. aureus* ATCC 29213, *Enterococcus faecalis* ATCC 29212, and *Streptococcus pneumoniae* ATCC49619 were used for internal quality control to ensure the accuracy and reproducibility of AST.

**Figure 1:**
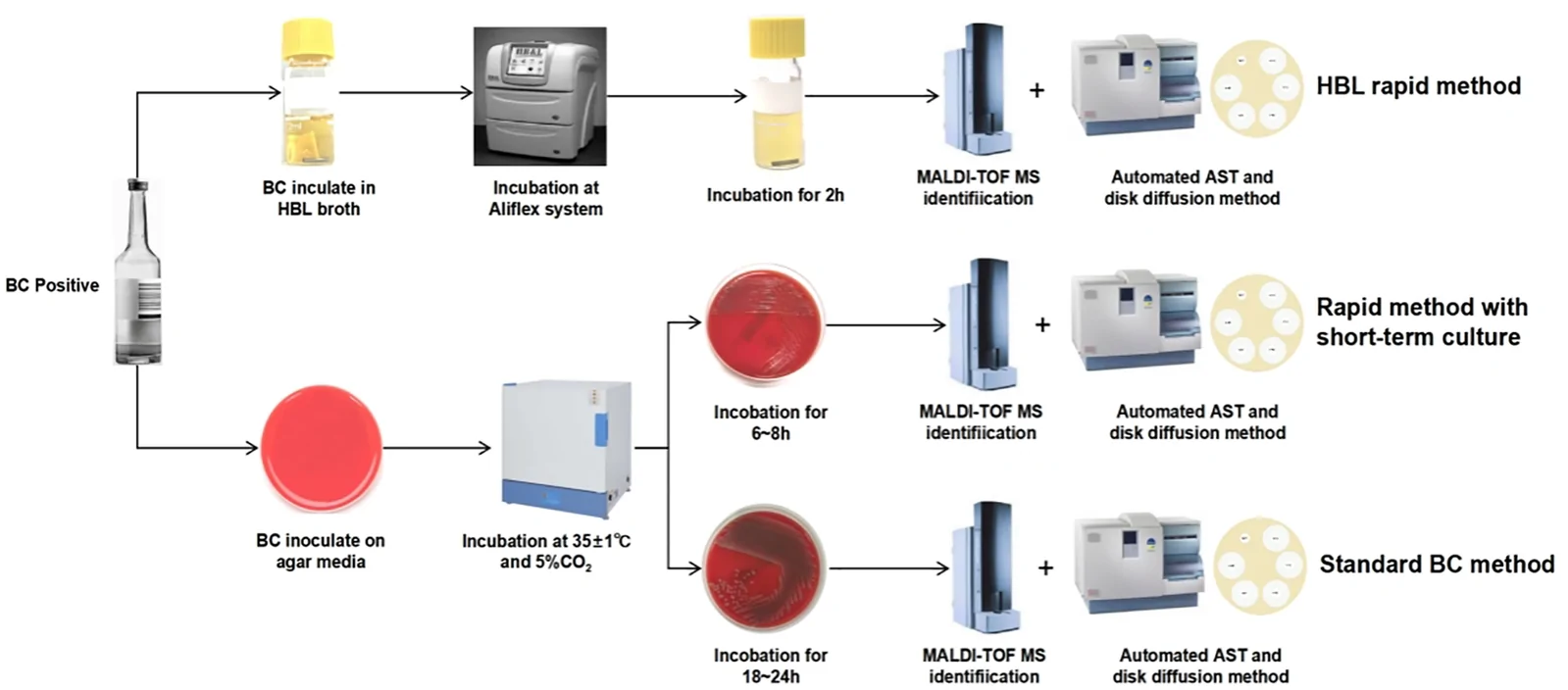
Three processing procedures of positive blood culture used in the study. The HBL rapid method and the rapid method with short-term culture were method with different short incubation. The standard blood culture method was based on colonies with overnight incubation. BC, blood culture.

### Rapid method with short-term culture

The rapid method involved short-term subculture of positive BC samples was performed as mentioned in **Figure 1**, with minor modifications to the conventional subculture workflow. Aliquots (500 μL) from positive BC bottles were directly subcultured onto Columbia blood agar plates with 5% sheep blood agar and MacConkey agar plates (Yihua Co., Ltd., Shanghai, China) and incubated at 35 ± 1 °C in a 5% CO_2_ atmosphere for a shortened period of 6–8 hours. Following this brief incubation, harvested bacterial colonies on the agar plates were used directly for immediate species identification via MALDI-TOF MS (Bruker Daltonics, Bremen, Germany). Concurrently, bacterial suspensions were prepared from the 6-8 hours short-term cultures, and AST was performed using the same species-specific methods as the standard BC method (Vitek 2 automated system or Kirby-Bauer disk diffusion method).

### HBL rapid method

The standard workflow of the HBL automated liquid culture system (Alifax, Polverara, Italy), a proprietary platform that detects bacterial growth via light-scattering technology, was further optimized and adapted for rapid processing of positive pediatric BC samples in this study (**Figure 1**), and this workflow reduced the TAT of bacterial culture. Briefly, a 100 μL aliquot from a positive BC bottle was inoculated into HBL enrichment broth vials, which were then loaded onto the HBL automated system for accelerated incubation at 35 ± 1 °C for 2 hours. The HBL system continuously and automatically monitors bacterial growth, and the optical density corresponding to a bacterial suspension of ≥0.5 McFarland (the minimum inoculum concentration required for reliable MALDI-TOF MS identification and AST) was recorded. After the 2 hours incubation, 500 μL of the enriched HBL broth was collected into a 1.5 mL Eppendorf tube for centrifugation at 12,000 × g for 5 min. The resulting supernatant was discarded, and the sediment was washed twice with sterile water. The final washed bacterial pellet was resuspended and spotted onto a MALDI-TOF MS Biotyper target plate for species-level identification. Additionally, the remaining enriched HBL broth was standardized to a 0.5 McFarland bacterial suspension and was used for AST analysis via Vitek2 automated susceptibility testing system (bioMérieux, Marcy l’ Étoile, France) or by disk diffusion method, as described above.

### Interpretation and comparison of AST results

The AST results of the two rapid methods were compared with those results obtained by using the standard AST method. For all AST assays, interpretive categories including susceptible[S], intermediate [I], and resistant [R] were determined in accordance with the Clinical and Laboratory Standards Institute (CLSI) 2024 guidelines. The interpretive criterion for tigecycline was based on the susceptibility breakpoints established by the Food and Drug Administration (FDA).

Agreement between the two rapid AST methods and the standard BC AST method was evaluated and categorized into the following categories: category agreement (CA), essential agreement (EA), very major error (VME; false susceptibility), major error (ME; false resistance), or minor error (mE; susceptible/resistant vs. Intermediate) (ISO, 2021; Tian et al., 2024). Then the Bias% (only for antimicrobials with at least 10 on-scale isolates) was also calculated according to ISO 20776-2: 2021. Some bacteria were intrinsically resistant to several antibiotics, and the results were not included for calculating EA and CA. A rapid AST method is deemed valid and clinically acceptable for a specific drug/microorganism combination when CA and EA are ≥90%, the VME rate is < 3% of the total resistant isolates, the ME rate is < 3% of the total susceptible isolates, and bias rates ≤ ± 30.0%, as indicated by ISO 20776-2: 2021.

### Evaluation of the TAT and statistical analysis

The TAT for each diagnostic workflow (two rapid methods and the standard method) was stratified and calculated for two distinct phases of blood culture processing: the pre-analytical phase and the post-positivity analytical phase. The pre-analytical phase was defined as the interval from the initiation of BC incubation in the Bactec FX400 system to the generation of a positive growth alert by the automated platform. The post-positivity analytical phase was defined as the total time required to complete all post-alert laboratory processes, including bacterial identification, AST, experimental result validation, and final report dissemination to the clinical care team. All statistical analyses were performed using GraphPad Prism version 5.0 (GraphPad Software, Inc., San Diego, CA, USA). Continuous variables (including TAT measurements) were presented as the mean ± standard deviation or the median. Categorical variables were expressed as numbers and percentages. The one-way ANOVA test was employed for comparing the differences in the continuous variables. Comparative analysis of categorical variables was carried out by the chi-square test. A value of *P* < 0.05 was considered statistically significant for all comparisons.

## Results

### Comparison of pathogen identification results of rapid methods

A total of 162 monobacterial positive blood culture (BC) samples were included in the final analysis, with differential processing of samples based on the time of BC positive alert as pre-specified in the study design. Specifically, 142 (87.7%) positive BC samples were processed in parallel using both the HBL rapid method and the rapid method with short-term culture, while the remaining 20 positive BC samples were only analyzed by the HBL rapid method, owing to the temporal constraints related to the BC positive alert time that precluded performance of the short-term culture method. All 162 monobacterial isolates recovered from the positive BC samples were subjected to pathogen identification, and the species distribution of the isolated pathogens is summarized in **Table 1**. Of the 162 total isolates, 108 (66.7%, 108/162) were Gram-negative bacteria, with the *Enterobacteriales* accounting for the vast majority (92.6%, 100/108) of this group. Within the *Enterobacteriales, E. coli* (34.3%, 37/108) and *Klebsiella pneumoniae* (27.8%, 30/108) were the most prevalent species. Gram-positive bacteria constituted the remaining 54 isolates (33.3%, 54/162), among which *S. aureus* (35.2%, 19/54) and *Enterococcus* spp. (33.3%, 18/54) were the predominant pathogens. Of *Enterococcus* spp., *Enterococcus faecalis* (50.0%, 9/18) and *Enterococcus faecium* (44.4%, 8/18) were commonly isolated. Other minor gram-positive bacterial genera included 5 *S. pneumoniae*, 5 *Streptococcus agalactiae*, and 7 other viridans streptococci. When compared with the standard BC method, the overall concordance rate of species-level identification results was 100% for the HBL rapid method (162/162) and the rapid method with short-term culture (142/142), which confirmed that rapid MALDI-TOF MS-based identification workflows can be reliably implemented for direct pathogen identification from positive BC samples without the need for prior pure colony isolation.

**Table 1:** Bacterial species identified by MALDI-TOF collected from BSI patients.

Bacterial species	Enrolled strains	HBL rapid method	Rapid method with short-term culture
	NO.	NO.	NO.
Gram-negative bacteria	108	108	92
*Escherichia coli*	37	37	34
*Klebsiella pneumoniae*	30	30	25
*Enterobacter cloacae* complex	10	10	9
*Serratia marcescens*	8	8	5
*Pseudomonas aeruginosa*	8	8	6
*Klebsiella oxytoca*	7	7	7
*Citrobacter freundii*	1	1	0
*Citrobacter koseri*	1	1	0
*Leclercia adecarboxylate*	1	1	1
*Salmonella* spp.	5	5	5
Gram-positive bacteria	54	54	50
*Staphylococcus aureus*	19	19	16
*Enterococcus faecalis*	9	9	9
*Enterococcus faecium*	8	8	8
Other *Enterococcus*	1	1	1
*Streptococcus pneumoniae*	5	5	5
*Streptococcus agalactiae*	5	5	5
Other viridans streptococcus	7	7	6

### Comparison of AST results of rapid methods

A total of 2051 and 1778 AST results were obtained from the HBL rapid method and the rapid method with short-term culture, respectively (**Table 2**). Compared with standard AST, the AST results of the two rapid methods yielded EA and CA values of > 95%. The rates of ME and VME were extremely low: 0.4% and 0.1% for the HBL rapid method, and 0.2% and 0.1% for the rapid method with short-term culture. Furthermore, the mE was also noted at 1.1% for the HBL rapid method and 1.0% for the rapid method with short-term culture. For gram-negative bacteria, the rates of EA and CA of the two rapid methods exceeded 90% for most pathogens, except for *Leclercia adecarboxylate*, representing 85.7% EA for the HBL rapid method and 78.6% EA and 85.7% CA for the rapid method with short-term culture, and *Citrobacter koseri*, representing 85.7% CA for the HBL rapid method. A few MEs and VMEs were observed in *K. pneumoniae* (ME: 1.2%; VME: 0.7%) and *Enterobacter cloacae* complex (ME: 0.8%) when using the HBL rapid method, whereas the MEs and VMEs of the rapid method with short-term culture were found in *E. coli* (ME: 0.2%; VME: 0.2%) and *K. pneumoniae* (ME:0.6%) (**Table 2**). For Gram-positive bacteria, the overall EA and CA values of both rapid methods were higher than 90%, with only MEs detected in two strains of *S. aureus* (ME: 0.7%) when using the HBL rapid method. Furthermore, the bias% analysis of the two rapid methods was also performed, and the bias rates of the HBL rapid method and the rapid method with short-term culture were -2.3% and -0.3%, respectively.

**Table 2:** Performance of the rapid methods compared to standard method for enrolled clinical isolates.

Bacterial species	HBL rapid method							Rapid method with short-term culture						
	No. of AST results	EA n/n (%)	CA n/n (%)	mE n/n (%)	ME n/n (%)	VME n/n (%)	Bias* (on-scale isolates)	No. of AST results	EA n/n (%)	CA n/n (%)	mE n/n (%)	ME n/n (%)	VME n/n (%)	Bias* (on-scale isolates)
Gram-negative bacteria														
*Escherichia coli*	518	507/518 (97.9)	513/518 (99.0)	5/518 (1.0)	–	–	-2.3% (37)	476	467/476 (98.1)	470/476 (98.7)	4/476 (0.8)	1/476 (0.2)	1/476 (0.2)	0.2% (34)
*Klebsiella pneumoniae*	420	399/420 (95.0)	408/420 (97.1)	4/420 (1.0)	5/420 (1.2)	3/420 (0.7)	0% (30)	350	337/350 (96.3)	344/350 (98.3)	4/350 (1.1)	2/350 (0.6)	–	-0.6% (25)
*Enterobacter cloacae* complex	130	122/130 (93.8)	124/130 (93.9)	5/130 (3.8)	1/130 (0.8)	–	1.4% (10)	117	112/117 (95.7)	115/117 (98.3)	2/117 (1.7)	–	–	–
*Serratia marcescens*	88	85/88 (96.6)	88/88 (100)	–	–	–	–	55	52/55 (94.5)	55/55 (100)	–	–	–	–
*Klebsiella oxytoca*	98	91/98 (92.9)	96/98 (98.0)	2/98 (2.0)	–	–	–	98	95/98 (96.9)	97/98 (99.0)	1/98 (1.0)	–	–	–
*Citrobacter freundii*	11	10/11 (90.9)	11/11 (100)	–	–	–	–	–	–	–	–	–	–	–
*Citrobacter koseri*	14	13/14 (92.9)	12/14 (85.7)	2/14 (14.3)	–	–	–	–	–	–	–	–	–	–
*Leclercia adecarboxylate*	14	12/14 (85.7)	13/14 (92.9)	1/14 (7.1)	–	–	–	14	11/14 (78.6)	12/14 (85.7)	2/14 (14.3)	–	–	–
*Pseudomonas aeruginosa*	96	91/96 (94.8)	93/96 (96.9)	3/96 (3.1)	–	–	–	72	66/72 (91.7)	69/72 (95.8)	3/72 (4.2)	–	–	–
*Salmonella* spp.	40	–	40/40 (100)	–	–	–	–	40	–	40/40 (100)	–	–	–	–
Gram-positive bacteria														
*Staphylococcus aureus*	285	269/285 (94.4)	282/285 (98.9)	1/285 (0.4)	2/285 (0.7)	–	-6.7% (19)	240	231/240 (96.3)	240/240 (100)	–	–	–	1.3% (16)
*Enterococcus faecalis*	72	–	72/72 (100)	–	–	–	–	72	–	71/72 (98.6)	1/72 (1.4)	–	–	–
*Enterococcus faecium*	64	–	64/64 (100)	–	–	–	–	64	–	64/64 (100)	–	–	–	–
Other *Enterococcus*	8	–	8/8 (100)	–	–	–	–	8	–	8/8 (100)	–	–	–	–
*Streptococcus pneumoniae*	60	15/15 (100)	60/60 (100)	–	–	–	–	60	15/15(100)	60/60 (100)	–	–	–	–
*Streptococcus agalactiae*	40	–	40/40 (100)	–	–	–	–	40	–	40/40 (100)	–	–	–	–
Other viridans streptococcus	56	7/7 (100)	56/56 (100)	–	–	–	–	48	6/6 (100)	48/48 (100)	–	–	–	–
Total	2014	1621/1796 (95.6)	1980/2014 (98.3)	23/2014 (1.1)	8/2014 (0.4)	3/2014 (0.1)	-2.3% (162)	1754	1392/1443 (96.5)	1733/1754 (98.8)	17/1754 (1.0)	3/1754 (0.2)	1/1754 (0.1)	-0.3% (142)

*Bias was evaluated when at least 10 on-scale MIC values were available; AST, antimicrobial susceptibility testing; CA, category agreement; EA, essential agreement; mE, minor error; ME, major error; VME, very major error.

For *Enterobacteriales*, the CA rates of two rapid methods were above 90% for most antimicrobial agents except for cefepime, which yielded a CA rate of 88.4% with the HBL rapid method (**Supplementary Table 1**). Furthermore, the VMEs of the HBL rapid method were reported for cefepime and trimethoprim-sulfamethoxazole in three strains of *K. pneumoniae.* Only one VME was detected for cefepime in *E. coli* when using the rapid method with short-term culture. For the HBL rapid method, one *K. pneumoniae* strain exhibited ME in five antimicrobial agents, and one *E. cloacae* complex strain showed ME in trimethoprim-sulfamethoxazole. In addition, for the rapid method with short-term culture, one *K. pneumoniae* strain was detected with MEs for cefepime and trimethoprim-sulfamethoxazole, and one *E. coli strain* had an ME for cefriaxone. Meanwhile, for *S. aureus*, the CA rates of two rapid methods were > 90% for most antimicrobial agents except for oxacillin (89.5%) in the HBL rapid method (**Supplementary Table 2**). The MEs were reported for oxacillin in two strains of *S. aureus.* The bias rates for selected antimicrobial agents of *Enterobacteriales* (excluding *Salmonella* spp) were -9.5%-4.2% in the HBL rapid method and -7.1%-2.4% in the rapid method with short-term culture, while the bias rates of the HBL rapid method and the rapid method with short-term culture for *S. aureus* were -26.3%-10.5% and -6.3%-12.5%, respectively (**Supplementary Tables 1**, **2**), which were considered acceptable.

### Comparison of TAT across the rapid methods and standard workflow

As depicted in **Table 3**, the mean time-to-positivity of all enrolled monobacterial positive BC samples was 12.22 hours. For pathogen identification, the mean TATs for the HBL rapid method were 21.8 ± 8.3 hours (95% CI: 20.5-23.0 hours), while that for the rapid method with short-term culture was 27.4 ± 8.1 hours (95% CI: 26.0-28.7 hours). Notably, both the rapid methods yielded significantly shorter identification TATs compared with the conventional standard method (*P* < 0.05), which requires overnight solid-media incubation before colony-based identification. Consistently, the mean TATs for AST in the HBL rapid method and the rapid method with short-term culture were 44.7 ± 9.1 hours (95% CI: 43.2~46.1 hours) and 45.9 ± 8.2 hours (95% CI: 44.6~47.3 hours), respectively, which were significantly reduced compared with the standard AST workflow (*P* < 0.05). Collectively, across all bacterial species evaluated in this study, the total combined TAT for pathogen identification and AST was shortened by approximately 24 hours for both rapid diagnostic approaches when compared with the standard BC workflow.

**Table 3:** Comparison of the TATs of three different methods for positive blood culture (hours).

Bacterial species	Time to positivity (Mean ± SD) (95% CI)	TAT from the initiation of BC testing to bacterial identification			TAT from the initiation of BC testing to AST results		
		HBL rapid method (Mean ± SD) (95% CI)	Rapid method with short-term culture (Mean ± SD) (95% CI)	standard method (Mean ± SD) (95% CI)	HBL rapid method (Mean ± SD) (95% CI)	Rapid method with short-term culture (Mean ± SD) (95% CI)	standard method (Mean ± SD) (95% CI)
Gram-negative bacteria	10.6 ± 5.0(9.6~11.6)	20.2 ± 6.7(19.0~21.5)	26.6 ± 6.5(25.3~28.0)	43.3 ± 8.2(41.7~44.9)	44.0 ± 7.8(42.5~45.5)	45.4 ± 6.6(44.1~46.8)	67.7 ± 11.1(65.6~69.8)
*Enterobacteriales*(exclud*ing Salmonella* spp.)	10.3 ± 5.1(9.2~11.3)	19.9 ± 6.7(18.6~21.3)	26.4 ± 6.9(24.9~27.9)	43.2 ± 8.4(41.5~44.9)	43.9 ± 8.0(42.3~45.5)	45.2 ± 6.9(43.7~46.7)	67.3 ± 11.0(65.1~69.6)
*Pseudomonas aeruginosa*	11.7 ± 3.6(8.7~14.8)	21.8 ± 7.6(15.5~28.2)	29.4 ± 2.0(27.2~31.5)	44.4 ± 9.0(36.8~51.8)	45.0 ± 8.8(37.7~52.3)	49.6 ± 2.4(47.1~52.1)	67.2 ± 9.9(58.9~75.4)
*Salmonella* spp.	15.3 ± 2.6(12.1~18.6)	23.6 ± 3.6(19.1~28.1)	26.5 ± 1.2(25.0~28.0)	43.5 ± 0.7(42.7~44.4)	44.0 ± 1.1(42.7~45.4)	44.0 ± 1.1(42.7~45.4)	76.2 ± 13.6(59.3~93.2)
Gram-positive bacteria	15.5 ± 8.6(13.0~17.8)	24.8 ± 10.2(22.0~27.6)	28.8 ± 10.2(25.9~31.7)	45.6 ± 12.1(42.3~48.9)	46.1 ± 11.2(43.0~49.1)	46.8 ± 10.5(43.9~49.8)	69.8 ± 9.1(67.3~72.3)
*Staphylococcus aureus*	19.7 ± 12.1(13.9~25.5)	28.4 ± 13.1(22.1~34.8)	33.5 ± 13.6(26.3~40.8)	46.8 ± 18.0(38.1~55.4)	48.2 ± 16.4(40.3~56.1)	49.9 ± 15.7(41.5~58.3)	72.8 ± 10.0(68.0~77.6)
*Enterococcus*	14.5 ± 4.3(12.3~16.6)	24.6 ± 6.6(21.3~27.8)	28.5 ± 5.8(25.6~31.4)	46.0 ± 5.9(43.1~49.0)	46.4 ± 5.8(43.5~49.3)	46.4 ± 5.8(43.5~49.3)	70.5 ± 6.4(67.3~73.7)
*Streptococcus*	11.8 ± 5.2(9.1~14.5)	21.0 ± 8.8(16.5~25.6)	24.4 ± 8.6(19.8~29.0)	43.9 ± 8.7(39.4~48.3)	43.3 ± 8.0(39.2~47.4)	44.2 ± 7.3(40.4~48.1)	65.7 ± 9.3(60.9~70.5)
All isolates	12.22 ± 6.8(11.2~13.3)	21.8 ± 8.3(20.5~23.0)	27.4 ± 8.1(26.0~28.7)	44.1 ± 9.7(42.6~45.6)	44.7 ± 9.1(43.2~46.1)	45.9 ± 8.2(44.6~47.3)	68.4 ± 10.5(66.8~70.0)

*TAT, turnaround time; AST, antimicrobial susceptibility testing.

## Discussion

Appropriate and timely administration of antimicrobial agents, on account of the bacterial species and AST results, is essential for clinical treatment of BSIs, which not only improves clinical outcomes and reduces mortality and length of hospital stay, but also maximally suppresses the development of bacterial resistance (Gajdos et al., 2025; Miller et al., 2024). To address this clinical challenge, novel methods for rapid identification and AST methods, including culture-based and culture-independent methods, have emerged to optimize the processing of positive BCs (Kim et al., 2024; Liborio et al., 2024). Even though several culture-independent methods for pathogen identification have been developed to greatly shorten the time to receive positive results, their clinical benefit remains unclear. Recently, culture-based methods were still the most established in routine clinical microbiology laboratories, which have been extensively evaluated over the years, are cheap, generally easy to perform, and available in all clinical microbiology laboratories (Bonaiuto et al., 2023; Liborio et al., 2024; Penven et al., 2025). In this study, we described two rapid methods for bacterial identification and AST and compared the clinical performance of the two rapid methods with the conventional BC workflow.

Rapid identification of microorganisms is performed by using MALDI-TOF MS, either directly on clinical samples or colonies following short-term culture on a plate. Previous studies have indicated that the accuracy rates of rapid identification at the species level were approximately 90%, or even as high as 99% (Dai et al., 2021; Huang et al., 2019; Zengin Canalp and Bayraktar, 2021). Meanwhile, multiple factors, including the type of clinical samples, polymicrobial growth, et al. can influence the accuracy of direct identification by MALDI-TOF MS. Blood cell components in blood culture are a critical factor affecting direct MALDI-TOF MS identification, and researchers have employed various chemical buffers such as Triton X-100 for improving the extraction efficiency of bacteria (Tai-Fen et al., 2025). For the HBL rapid method, 100 μl positive BC medium was subcultured into HBL enrichment broth and centrifugation with repeated washing was performed to get rid of the blood cells. Alternatively, another rapid process based on short-term incubation has been proposed for identifying bacterial pathogens, which can eliminate the need for centrifugation and additional chemicals for bacterial protein extraction. Some studies have reported that the species-level identification concordance rate was 90.9% after 6 hours of incubation and 80.6% after 4 hours of incubation, with the optimal incubation time potentially depending on the bacterial species (Hong et al., 2014; Tsai et al., 2021). In this study, an incubation time of 6-8 hours was selected for all bacterial isolates to achieve rapid identification of positive BCs. In addition, it should also be taken into consideration that the presence of polymicrobial growth in positive BCs might lead to misidentification and thus affect the accuracy of direct MALDI-TOF MS identification, which necessitates the use of supplementary methods including Gram staining and culture growth in solid media. Therefore, this study enrolled positive BCs with monomicrobial growth for rapid identification to reduce the impact of polymicrobial growth, and the identification accuracy rates of two rapid methods by using MALDI-TOF MS were both 100%, which revealed that rapid MALDI-TOF MS-based identification workflows can still be reliably implemented for direct pathogen identification from positive BC samples without the need for prior pure colony isolation.

Regarding the use of antimicrobial agents based on the minimum inhibitory concentration of bacteria isolated from positive BCs, new rapid AST methods involved phenotypic testing, including the Ovella FAST system (Penven et al., 2025) and Alfred^60^AST (Mantzana et al., 2021), and genetic testing, including Xpert Carba-R (Seo et al., 2022), are currently recommended to improve the timeliness of infection treatment. The HBL automated system provided AST directly on positive BCs via light scattering, and an agreement rate of above 90% had been reported in recent studies (Mantzana et al., 2021; Van den Poel et al., 2020). In the present study, we conducted 2-hour incubation of BCs by the HBL system and then performed AST, and results exhibited excellent performance compared with the standard method, with an EA rate of 95.6%, a CA rate of 98.3%, a VME rate of 0.1%, a ME rate of 0.4%, a mE rate of 1.1%, and a bias rate of -2.3%, which consistent with those of previous reports. The VMEs were limited to cefepime (1/30) and trimethoprim- sulfamethoxazole (2/30) for *K. pneumoniae*. On the other hand, the processing of AST was conducted for the colonies obtained from 6-8 hours of incubation of positive BCs in blood agar media. The present research showed that the rates of EA, CA, VME, ME, mE, and bias of the rapid method with short-term culture were 96.5%, 98.8%, 1.0%, 0.2%, 0.1%, and -0.3%, respectively, which were consistent with those reported in other studies (Ha et al., 2018; Morecchiato et al., 2025). Additionally, the VME was limited to cefepime in *E. coli* (1/37), while the MEs were detected for cefepime (1/30) and trimethoprim-sulfamethoxazole (1/30) for *K. pneumoniae* and cefriaxone (1/37) for *E. coli*. Overall, both the rapid methods presented excellent results with low error percentages (mE, ME, VME) meeting the performance standards of the U.S. FDA (mE < 3%, VME < 1.5%), which can be routinely carried out in clinical microbiology laboratories.

From the viewpoint of the TAT of positive BCs, previous studies have shown that the rapid methods integrating microbial identification and AST directly from the BC bottle provided a time saving of at least 24 hours compared with the standard method, which relies on overnight subculture-based routine workflows (Anton-Vazquez et al., 2019; Bonaiuto et al., 2023). However, limited research has been conducted on rapid identification and AST of positive BCs in pediatric populations. The present study showed that the mean TATs of the two rapid methods were 44.7hours and 45.9 hours, respectively, which were shorter than that of the standard method (68.4 hours), which was consistent with previous findings (Bonaiuto et al., 2023; Dai et al., 2021). Given the limited range of antimicrobial agents available for children, inappropriate antibiotic use may directly result in missed treatment opportunities and failure to control the disease in pediatric patients. Therefore, timely bacterial species identification and AST may greatly assist clinicians in selecting appropriate antimicrobial agents in children, thereby reducing the mortality rate associated with BSIs (Halperin et al., 2025). Notably, the implementation of these rapid methods allows the final AST report for positive BCs to be typically available on the second day after the BCs are flagged as positive.

This study has several limitations. Firstly, the present study is a single-center retrospective design, and regional epidemiological discrepancies may affect the extrapolation of our pathogen and resistance data to other medical centers. Secondly, owing to the difference in the time to positivity of BCs, a time-dependent testing algorithm for samples (162 samples using both the HBL rapid method and 142 by the rapid method with short-term culture) was conducted; however, this asymmetry in the generalizability of the performance estimates potentially impacted the results of the short-term culture method. Thirdly, the absence of broth microdilution data is one of the important limitations in this study. Due to isolate viability or storage constraints, we could not retrospectively assess the clinical performance of two rapid methods with the broth microdilution method, which can reflect that the validation reported is comparative rather than absolute in nature, and that true accuracy against a gold standard remains undemonstrated. Then, the discrepancy analysis during testing was not further confirmed by using additional reference methods.

## Conclusion

In conclusion, the concordance rates between the two rapid methods and the conventional standard method presented in this study were high (> 90%), demonstrating that these rapid methods provide a reliable and accurate approach for the identification and AST of positive BCs. Furthermore, the two rapid methods significantly shorten the processing time of positive BCs, resulting in a reduction of TAT of nearly 24 hours. Clinical microbiologists should select a simple and effective rapid method to optimize the positive BC processing procedures, which is conducive to the rapid optimization of antibiotic use in clinical practice.

## Data Availability

The original contributions presented in the study are included in the article/**Supplementary Material**. Further inquiries can be directed to the corresponding author.

## Supplementary material

Supplementary Table 1 AST results of the rapid methods of Enterobacteriales (exclud*ing Salmonella* spp.).

Supplementary Table 2 AST results of the rapid methods of *Staphylococcus aureus.*
