# Supplementary material for: Performance comparison and clinical validation of two rapid direct identification and antimicrobial susceptibility testing approaches for pathogens isolated from positive blood cultures in children
Source: Front Cell Infect Microbiol. 2026 Sep 17;16:1861487. doi: 10.3389/fcimb.2026.1861487 (PMC13626878; doi:10.3389/fcimb.2026.1861487)
Supplement: Supplementary file 1 [file Table1.docx]

**TABLE S1** AST results of the rapid methods of Enterobacteriales (exclude *Samlonella* spp.)

| Antimicrobial agents | HBL rapid method | | | | | | | Rapid method with short-term culture | | | | | | |
| --- | --- | --- | --- | --- | --- | --- | --- | --- | --- | --- | --- | --- | --- | --- |
|  | No. of AST results | EA  n (%) | CA  n (%) | mE  n (%) | ME  n (%) | VME  n (%) | Bias*  (on-scale  isolates) | No. of AST results | EA  n (%) | CA  n (%) | mE  n (%) | ME  n (%) | VME  n (%) | Bias*  (on-scale  isolates) |
| Amoxicillin-clavulanate | 76 | 75(98.7) | 76(100) | - | - | - | -6.3%(76) | 67 | 66(98.5) | 67(100) | - | - | - | 0%(67) |
| Piperacillin-tazobactam | 95 | 92(96.8) | 94(98.9) | - | 1(1.1) | - | -1.1(95) | 81 | 78(96.3) | 79(97.5) | 2(2.5) | - | - | 1.2%(81) |
| Cefuroxime | 86 | 81(94.2) | 85(98.8) | - | 1(1.1) | - | -1.1%(86) | 76 | 74(97.4) | 76(100) | - | - | - | 0(76) |
| Cefoxitin | 86 | 84(97.7) | 86(100) | - | - | - | 0%(86) | 76 | 75(98.7) | 76(100) | - | - | - | 1.2%(76) |
| Ceftazidime | 95 | 93(97.9) | 93(97.9) | 1(1.1) | 1(1.1) | - | 0%(95) | 81 | 80(98.8) | 80(98.8) | 1(1.2) | - | - | 0%(81) |
| Cefriaxone | 95 | 92(96.8) | 94(98.9) | - | 1(1.1) | - | -2.1%(95) | 81 | 78(96.3) | 80(98.8) | - | 1(1.2) | - | -1.2%(81) |
| Cefperazone-sulbactam | 95 | 93(97.9) | 90(94.7) | 5(5.3) | - | - | -5.3%(95) | 81 | 81(100) | 79(97.5) | 2(2.5) | - | - | -1.2%(81) |
| Cefepime | 95 | 86(90.5) | 84(88.4) | 9(9.5) | 1(1.1) | 1(1.1) | -9.5%(95) | 81 | 75(92.6) | 74(91.4) | 5(6.2) | 1(1.2) | 1(1.2) | -7.1%(81) |
| Ertapenem | 95 | 89(93.7) | 93(97.9) | 2(2.1) | - | - | 0%(95) | 81 | 77(95.1) | 79(97.5) | 2(2.5) | - | - | 1.2%(81) |
| Imipenem | 95 | 91(95.8) | 95(100) | - | - | - | 0%(95) | 81 | 77(95.1) | 81(100) | - | - | - | 2.4%(81) |
| Amikacin | 95 | 90(94.7) | 93(97.9) | 2(2.1) | - | - | 4.2%(95) | 81 | 81(100) | 81(100) | - | - | - | 0%(81) |
| Levofloxacin | 95 | 95(100) | 95(100) | - | - | - | 0%(95) | 81 | 81(100) | 80(98.8) | 1(1.2) | - | - | -1.2%(81) |
| Tigecycline | 95 | 87(91.6) | 95(100) | - | - | - | -2.1%(95) | 81 | 72(88.9) | 81(100) | - | - | - | -4.7%(81) |
| Trimethoprim-sulfamethoxazole | 95 | 91(95.8) | 92(96.8) | - | 1(1.1) | 2(2.1) | -2.1%(95) | 81 | 79(97.5) | 80(98.8) | - | 1(1.2) | - | 2.4%(81) |
| Total | 1293 | 1239(95.8) | 1265(97.8) | 19(1.5) | 6(0.5) | 3(0.2) | -2.1%(95) | 1110 | 1074(96.8) | 1093(98.5) | 13(1.2) | 3(0.3) | 1(0.1) | -0.5%(81) |

*Bias was evaluated when at least 10 on-scale MIC values were available; AST: antimicrobial susceptibility testing; CA: category agreement; EA: essential agreement; mE: minor error; ME: major error; VME: very major error.

**TABLE S2** AST results of the rapid methods of *Staphylococcus aureus*

| Antimicrobial agents | No. of AST results | HBL rapid method | | | | | | No. of AST results | Rapid method with short-term culture | | | | | | |
| --- | --- | --- | --- | --- | --- | --- | --- | --- | --- | --- | --- | --- | --- | --- | --- |
|  |  | EA  n (%) | CA  n (%) | mE  n (%) | ME  n (%) | VME  n (%) | Bias*  (on-scale  isolates) |  | EA  n (%) | CA  n (%) | mE  n (%) | ME  n (%) | VME  n (%) | Bias*  (on-scale  isolates) |  |
| Penicillin | 19 | 19(100) | 19(100) | - | - | - | 0%(19) | 16 | 16(100) | 16(100) | - | - | - | 6.3%(16) | |
| Oxacillin | 19 | 13(68.4) | 17(89.5) | - | 2(10.5) | - | -26.3%(19) | 16 | 14(87.5) | 16(100) | - | - | - | -6.3%(16) | |
| Ceftaroline | 19 | 19(100) | 19(100) | - | - | - | 10.5%(19) | 16 | 16(100) | 16(100) | - | - | - | 12.5%(16) | |
| Gentamicin | 19 | 19(100) | 19(100) | - | - | - | 0%(19) | 16 | 16(100) | 16(100) | - | - | - | 0%(16) | |
| Levofloxacin | 19 | 14(73.7) | 19(100) | - | - | - | -26.3%(19) | 16 | 12(75.0) | 16(100) | - | - | - | 6.3%(16) | |
| Moxifloxacin | 19 | 19(100) | 19(100) | - | - | - | 0%(19) | 16 | 16(100) | 16(100) | - | - | - | 0%(16) | |
| Erythromycin | 19 | 18(94.7) | 18(94.7) | 1(5.3) | - |  | -10.5%(19) | 16 | 16(100) | 16(100) | - | - | - | 0%(16) | |
| Clindamycin | 19 | 18(94.7) | 19(100) | - | - | - | -5.3%(19) | 16 | 16(100) | 16(100) | - | - | - | 0%(16) | |
| Linezolid | 19 | 19(100) | 19(100) | - | - | - | -5.3%(19) | 16 | 16(100) | 16(100) | - | - | - | 6.3%(16) | |
| Daptomycin | 19 | 18(94.7) | 19(100) | - | - | - | -5.3%(19) | 16 | 16(100) | 16(100) | - | - | - | 0%(16) | |
| Teicoplanin | 19 | 19(100) | 19(100) | - | - | - | 0%(19) | 16 | 16(100) | 16(100) | - | - | - | 0%(16) | |
| Vancomycin | 19 | 17(89.5) | 19(100) | - | - | - | -10.5%(19) | 16 | 13(81.3) | 16(100) | - | - | - | -6.3%(16) | |
| Tigecycline | 19 | 19(100) | 19(100) | - | - | - | 0%(19) | 16 | 16(100) | 16(100) | - | - | - | 0% | |
| Rifampicin | 19 | 19(100) | 19(100) | - | - | - | 0%(19) | 16 | 16(100) | 16(100) | - | - | - | 0%(16) | |
| Trimethoprim-sulfamethoxazole | 19 | 19(100) | 19(100) | - | - | - | 0%(19) | 16 | 16(100) | 16(100) | - | - | - | 0%(16) | |
| Total | 285 | 269(94.4) | 282(98.9) | 1(0.4) | 2(0.7) | - | -6.7%(19) | 240 | 231(96.3) | 240(100) | - | - | - | 1.3%(16) | |

*Bias was evaluated when at least 10 on-scale MIC values were available; AST: antimicrobial susceptibility testing; CA: category agreement; EA: essential agreement; mE: minor error; ME: major error; VME: very major error.
